# Abalones at risk: A global Red List assessment of *Haliotis* in a changing climate

**DOI:** 10.1371/journal.pone.0309384

**Published:** 2024-12-23

**Authors:** Howard Peters, Gina M. Ralph, Laura Rogers-Bennett

**Affiliations:** 1 Department of Environment and Geography, University of York, Heslington, York, United Kingdom; 2 IUCN Marine Biodiversity Unit, Department of Biological Sciences, Old Dominion University, Norfolk, Virginia, United States of America; 3 Bodega Marine Laboratory, California Department of Fish and Wildlife and University of California Davis, Bodega Bay, California, United States of America; Fisheries and Oceans Canada, CANADA

## Abstract

There is increasing awareness that marine invertebrates such as abalones are at risk from the combined stressors of fishing and climate change. Abalones are an important marine fishery resource and of cultural importance to Indigenous and non-Indigenous people. A highly priced marine delicacy, they are inherently vulnerable: individuals are slow-growing and long-lived and successful reproduction requires dense assemblages. However, their global conservation status is poorly understood. Using IUCN Red List methodology, we assessed the extinction risk to all 54 species of abalone (genus *Haliotis*). Of the 21 fished commercially for human consumption either now and/or in the past, 15 (71.43%) are classified as threatened, i.e., those identified as Critically Endangered, Endangered or Vulnerable. Of the 33 unexploited species, only five (15.15%) are so classified, making exploited species over four times more likely to face extinction, underscoring the impact of fishing on abalones already confronting a changing climate. The highest concentration of threatened species occurs along the North American Pacific coast. Here six of the seven species have been exploited, yet despite years of fishery closures with exemptions only in Alaska and Mexico, all are categorised as threatened. Climate driven stressors have led to mass mortalities, with competition from sea urchins and disease, aggravated by harmful algal blooms. In Australia the picture is mixed despite robust stock management, with some regions experiencing mass mortalities from marine heatwaves and viral spread. Poaching has reached its apogee in South Africa, where organised criminal gangs have reduced the legal fishery of *Haliotis midae*, ‘perlemoen’ almost to a footnote, accompanied by widespread recruitment failure. In response, the authorities have focused on abalone ranching and stock enhancement. In Japan, with a long history of abalone fishing, wild stocks are routinely supplemented with hatchery-bred juveniles. Collaboration between restoration aquaculture and fisheries, including sea urchin control and kelp restoration, offers hope for rebuilding stocks against a backdrop of escalating environmental stressors.

## Introduction

Extinction risk for species in marine environments was once considered nearly impossible owing to their perceived abundance and the belief that marine species had unlimited fecundity [1], but today it has become reality [2]. Marine molluscs such as the eelgrass limpet (*Lottia alveus*), horned snail (*Neoplanorbis carinatus*), Chinese periwinkle (*Littoraria flammea*) and rocky shore limpet (*Lottia edmitchelli*) have all become extinct [3, 4], with white abalone (*H*. *sorenseni*) now also on the verge of extinction [5]. Given the combined impacts of human exploitation and a changing climate, the risk of marine extinctions is now universally accepted within policy arenas with overexploitation as the principal driver [2]. As human populations increase, overfishing, both legal and illegal, is affecting the abundance and productivity of marine taxa [6–8]. Deficiency of data, that would otherwise flag population change, can result in overfishing even of common species [9] and rare species frequently lack data on abundance [10]. For example, the giant clam (*Tridacna gigas*) has been fished out of extensive areas it once inhabited including within Fiji, Guam and New Caledonia and is listed Vulnerable on the IUCN Red List [11]. Similarly, queen conch (*Lobatus gigas*) has been overfished in many parts of the Caribbean [12]. Overfishing of California’s once abalone-rich nearshore waters has resulted in the serial depletion of abalone stocks in a gradient from the most popular to the least popular species, and nearest to furthest from port [13].

Effects of overfishing are exacerbated by a variety of human-mediated environmental stressors most notably elevated levels of CO_2_. Increases in CO_2_ resulting from the combustion of fossil fuels, both intensifies ocean warming and leads to ocean acidification, where excess CO_2_ combines with seawater and if unchecked can impede calcification in molluscs and other marine invertebrates [14]. Changes in climate are leading to rising sea levels, with warming oceans intensifying the incidence and severity of hurricanes, inundating low-lying coastal zones, and displacing human populations [15]. Climate-driven marine heatwaves (MHWs) together with pollution and other stressors are causing catastrophic declines in abundance of marine taxa, accelerating the loss of biodiversity and changes to marine food-webs [16, 17]. Further declines in foundation species can lead to transitions to alternate states that are far less species rich. In coral reef systems, declines in corals due to a combination of overfishing, pollution and disease have precipitated the transition from coral dominated systems to macroalgal systems with the concurrent loss of biodiversity [18]. Similarly, MHWs have triggered the loss of kelp forests, transitioning to sea urchin barrens—large areas with high sea urchin populations, no macroalgae and little or no abalones [19–21]. In Australia, warming waters have led to the expansion of the sea urchin *Centrostephanus rodgersii* into waters that were previously too cold for these warm water echinoderms [22, 23]. Disease outbreaks, thought to be exacerbated by warming, are also a major cause of decline in marine populations and biodiversity [24, 25]. Black abalone (*H*. *cracherodii*) populations, once abundant in the intertidal habitats in southern California, have been decimated by overfishing and disease throughout the Channel Islands [26] with black abalone now listed under the US Endangered Species Act. Clearly there are tipping points [27] beyond which many marine ecosystems collapse [28].

Approximately 46 000 marine mollusc species have been recorded with possibly a further 150 000 waiting to be identified and described, many from the deep seas [29]. Despite their importance to biodiversity, the marine food web and as a fishery resource, their conservation status is barely known. Mollusca is the phylum most impacted by extinction with more than 300 species listed by IUCN Red List as having become extinct and although marine species may be less prone to extinction than terrestrial or freshwater species, the true number may be considerably more than this [30]. However, despite their ubiquity, the IUCN Red List of Threatened Species, the world’s leading database on species’ extinction risk, includes just 1886 assessments of marine molluscs including 1047 from the taxonomic class Gastropoda, although 60% of these are of one family—Conidae, the cone snails [31, 32]. To place this in context, 74% of all fishes, 85% of reptiles, 90% of mammals, 92% of amphibians and 100% of birds have been assessed (www.iucnredlist.org/resources/summary-statistics).

In 2003 and 2006 respectively, just two abalone species had been assessed for the Red List: *H*. *cracherodii* and *H*. *kamtschatkana*, both from Pacific North America. We assessed the current status and extinction risk of all 54 abalone species globally using IUCN Red List criteria. We explore each species’ distribution, current and projected threats from exploitation, poaching, environmental stress and ecological disruption. Our aim is to provide data in support of conservation measures for those species at the greatest risk of extinction over the short to medium term to inform climate smart restoration planning and action.

### Abalones

Abalones are of the family Haliotidae, with a single genus, *Haliotis*, the etymology of which is from the Greek for sea-ear, reflecting its aural form. The name abalone derives from the American Spanish *abulón* which in turn is a corruption of *aulon* in Rumsen, an Indigenous language, now extinct, of Monterey Bay, California [33]. Abalones first appear in the Late Cretaceous although the fossil record is poor with extended periods without evidence until their reappearance in the late Eocene, and then with greater frequency from the Late Miocene to the present [34]. Abalones occur primarily in temperate regions although some species are found in tropical seas. They most often live in shallow, rocky, sublittoral waters between the subtidal and 30 m depth, although there are exceptions: *H*. *pourtalesii* for example, occurs from the Carolinas south to Brazil at 35 m to 350 m depth [34, 35]. Abalones have been important to Indigenous peoples for millennia [36]. Today they are of national and international commercial importance, primarily as a food delicacy but also for the exquisite nacre lining their shells which is used for jewellery and mother-of-pearl inlay in marquetry. Although it is normally the larger species that are consumed, there are instances of smaller species also finding favour including *H*. *ovina*, the sheep’s ear abalone at 80 mm and *H*. *asinina*, the donkey’s ear abalone at 100 mm. These tropical species, known as cocktail-size abalones, occur across the Indo West Pacific [37]. Abalones also fulfil an important ecological function in grazing on algae enabling open areas of benthic crustose coralline pavement suitable for the recruitment of abalones and other species [38].

Although abalones are generally small to medium in size, i.e. less than 100 mm, some are particularly large such as *H*. *gigantea* at 233 mm, with the current record held by a red abalone (*H*. *rufescens*) from Pacific North America at 313 mm taken by John Pepper [39]. Depending on species, abalones may be oval to rounded with generally three or four whorls and a small flat spire. Shells range from smooth to those with protruding sculptured elements including ridges, bumps and striations [34]. The most distinguishing feature of all abalone species is a row of multi-functional holes around the margin of the anterior, used in the respiration of water from the gills, release of faeces, and expulsion of gametes into the water column [34]. The first hole appears in juveniles of 1 to 3 mm length and then they increase in number, with new ones appearing from the anterior margin as the abalone develops. Older holes towards the posterior are sealed by the mantle such that only the most recent holes are open at any point in the abalone’s life. The number of open holes does not seem to be consistent to any species but are typically five or six but sometimes more [34]. Abalone shell has exceptional tensile strength [40, 41] with practical applications in materials research including bone tissue engineering [42].

Although abalones are found across the globe (excluding polar regions), there are some notable voids. For example, although occurring along the Pacific coast of North America from Alaska to Baja California, Mexico, on the Atlantic coast, a paucity of suitable rocky habitat probably accounts for their absence as far south as the Carolinas. Even then, just a single species, *H*. *pourtalesii*, occurs thereafter south along the Atlantic coast of Central and South America. Apart from the Galápagos Islands and Cocos Island of Costa Rica, the entire length of coastline of Pacific South and Central America is similarly devoid of abalones despite the presence of rocky shorelines that would seem to offer suitable habitat. Areas of distribution vary widely between species, with some, such as *H*. *rubiginosa*, found only in the confined waters of Lord Howe Island in the Tasman Sea off the east coast of Australia. Some species, however, occur across the length of the Indian Ocean and Western Pacific, such as *H*. *ovina*, found from the Maldives in the west to the Society Islands in French Polynesia in the east, a distance of approx. 14 900 km.

Abalones are broadcast spawners with separate males and females. They typically reach maturity within five to eight years and release their gametes into the water column on a cue standard to each species. These cues may include a sudden variation in sea-surface temperature (SST) or tidal height [43], lunar cycle, and even typhoon events, as in the case of *H*. *diversicolor* [44]. Once released, for successful fertilisation the eggs must rapidly encounter high densities of sperm. This demands closely packed assemblages of animals for reproductive success. Experiments with greenlip abalone (*H*. *laevigata*) of South Australia have shown that when the distance between male and female is greater than two metres fertilization failure ensues [45]. Known as the Allee Effect, this is a common biological phenomenon [46] that positively correlates population density with individual fitness. It is also one of the most significant influencers in the likelihood of recovery of a population decimated by overfishing or mass mortality, where remnant populations may become too widely dispersed and the individuals eventually age and die without reproducing successfully. This has been demonstrated in the case of white abalone (*H*. *sorenseni*), where a general model was developed that determined that too few remain for successful reproduction throughout its range in the wild [5].

The larvae of abalones are lecithotrophic and form the first shell, the protoconch, while still planktonic. Settlement of the larvae occurs after one or two weeks and is induced by a chemical signal from crustose coralline algae (CCA) [47, 48]. Abalones are mainly sedentary, although some species move more than others in search of food. Young juvenile abalones typically forage and feed at night returning during the daytime to their cryptic habitat under boulders and in crevices. Older juveniles and adults may have a home site, or remain in one place to capture drift algae or longer strands of attached algae [49]. Although most species feed exclusively on algae, a few, including those occurring in Australian waters, also consume seagrass and their epiphytes [50]. Abalones’ exceptionally powerful adhesion to rocky surfaces through a pedal foot enables them to occupy environments with strong currents and wave activity. Their remarkable ability results from setae and nanoscale terminations enabling capillary and van der Waals forces to seal the interface between foot and rock on a range of surface conditions [51]. When disturbed, abalones retract their epipodium and tentacles that line the margin of the shell and clamp the shell fast to the rock with their foot, becoming virtually immovable.

In common with other molluscs, abalone haemolymph does not coagulate, and so removal must be accomplished without damage to avoid death, although trials have shown that a degree of survival is possible [52]. To achieve this, abalones have to be quickly hand-levered by a diver using a specially rounded abalone iron. In deeper water, SCUBA or hookah i.e., surface-supplied air, are used to fish for abalones. Once harvested, abalones may be sold whole, shucked, canned or dried. Although most of the animal can be eaten, the foot muscle with the end trimmed is preferred, but it requires slicing and tenderising to make it palatable and ready for cooking. The abalone fishery worldwide peaked in the 1970s at nearly 20 000 mt but by 2020 the wild fishery had collapsed to 4500 mt [53, 54]. Today, aquaculture production has far surpassed wild abalone fisheries accounting for more than 98% of the total abalone market [54], including regions such as Chile which lack naturally occurring wild abalones. In very few regions wild fisheries still dominate, with wild-taken abalones being the preferred option in some countries, especially for ceremonial occasions.

Abalones reflect many of the conservation issues confronting shallow-water marine taxa: 1) fished in large numbers for human consumption with the threat of overexploitation by both legal and illegal means; 2) necessity for closely packed assemblages to ensure fertilisation success that becomes vulnerable through overfishing; 3) exposure to the increasing prevalence of MHWs resulting from a changing climate directly impacting their mortality and/or resulting in loss of algal food resource; 4) reduction in habitat through invasive species in particular sea urchins that compete for food; and 5) vulnerability to surface-borne pollutants, including runoff from industry and agriculture, pathogen escapes from aquaculture facilities, and anti-fouling agents used on hulls of vessels. By using the standard assessment methodology developed by the IUCN Red List, the objective of our research was to identify those species whose conservation status may be affected by these and other threats. The detailed results summarised here are published and freely available on www.iucnredlist.org.

## Methods

The IUCN Red List is the world’s leading resource for describing the global conservation status of animals, plants and fungi, and uses a standard methodology to classify species into one of nine categories supported by a codified set of criteria. The assessment process examines the impact of threats on species, but also includes the effect of any recovery programmes. For each species, data are collected using a standard format together with images, maps and supporting documentation [55].

Before developing the database for our global abalone assessment, we referenced the World Register of Marine Species (www.marinespecies.org) to create a taxonomic list of all 54 species of *Haliotis* which we loaded into the Species Information System (SIS), the online assessment tool of the IUCN Red List of Threatened Species that acts both as a repository for the data and as a ‘calculator’, described below, to determine the categories and criteria that are central to establishing the level of threat.

### Categories and criteria

Each species assessed under the procedures of the IUCN Red List is assigned a category (Table 1) that assigns the level of risk, provided sufficient data are available to make such determination [55]. There are nine categories, of which the three most at risk (Critically Endangered, Endangered and Vulnerable) are collectively named as threatened categories. The assessment process is common to all species, whether animals, plants or fungi, and the category is assigned on the basis of five criteria (A-E) (Table 2) established from research. The criteria code(s) appropriate to a taxon are determined from those elements that influence its status, e.g. population decline, changes in area of distribution, etc. selected from a series of tables and sub-tables. For example: Criterion A has four divisions: A1: population reduction observed, estimated, inferred, or suspected in the past where the causes of the reduction are clearly reversible AND understood AND have ceased. Criterion A2 is as per A1 but when the causes may not be reversible or understood or ceased. Criterion A3 refers to future projections while A4 refers to both the past and future. These population reductions require to be further supported by one or more elements from a sub-table, i.e. (a) from direct observation; (b) an index of abundance appropriate to the taxon; (c) a decline in area of occupancy (AOO), extent of occurrence (EOO) and/or habitat quality; (d) actual or potential levels of exploitation; and/or (e) effects of introduced taxa, hybridization, pathogens, pollutants, competitors or parasites. Other criteria codes (B-E) are structured in a similar fashion being summarised at www.iucnredlist.org/resources/summary-sheet. In fulfilling such categorisation, it is assessed as threatened, i.e. CR, EN or VU, depending on the percentage reduction over the longer of ten years or three generations.

**Table 1:** The nine categories.

Category	Code	Test
Extinct	EX	There is no reasonable doubt that the last individual has died measured over a time frame appropriate to the taxon’s life cycle and life form
Extinct in the Wild	EW	The taxon is known only to survive in cultivation, in captivity or as a naturalized population(s) well outside the past range
Critically Endangered	CR	The taxon meets any of the criteria A to E for Critically Endangered, and it is therefore considered to be facing an extremely high risk of extinction in the wild
Endangered	EN	As CR but considered to be facing a very high risk of extinction in the wild
Vulnerable	VU	As CR but considered to be facing a high risk of extinction in the wild
Near Threatened	NT	The taxon has been evaluated against the criteria but does not qualify for CR, EN or VU now, but is close to qualifying for or is likely to qualify for a threatened category in the near future
Least Concern	LC	The taxon has been evaluated against the criteria and does not qualify for CR, EN, VU or NT. Widespread and abundant taxa are included in this category
Data Deficient	DD	There is inadequate information to make a direct, or indirect, assessment of the taxon’s risk of extinction based on its distribution and/or population status
Not Evaluated	NE	The taxon has not yet been evaluated against the criteria

**Table 2:** The five criteria.

Criterium	Parameter
A	Population size reduction, i.e., trend
B	Decline and/or fragmentation of extent or area of occupancy or habitat
C	Small population size with a continuing decline or fluctuation in mature individuals
D	Very small or restricted population
E	Quantitative analysis with a probability of extinction over a defined period

### Method of assessment

We followed IUCN Red List standards that requires all assessments be based on data that are currently available for taxa across their entire global range. The primary means of collecting data on abalone species was through comprehensive literature searches in scientific journals and fishery and trade publications, together with specialist reports on regional catch statistics, wildlife crime, aquaculture, abalone ranching and restoration projects. National and regional fishery authorities periodically publish abalone catch quotas and stock assessments and, together with fishery statistics and analyses of catch data, provided essential data in determining population trends. We consulted malacologists, fisheries managers, and international dealers in mollusc shells, as well as marine conservationists and experts in illicit wildlife trade through telephone and online conference calls, workshops, email, and web-based discussions, and analysing monitoring datasets to determine population trends (www.iucnredlist.org/assessment/process). All data was transferred to the IUCN online assessment database–SIS.

To ensure all species’ assessments use the same classification system for threats, habitats, etc. SIS uses a standardized format for managing assessment data, in addition to ensuring taxonomic integrity. This enforces consistency in application and facilitates the evaluation process. It allows the user to enter biological, population, range, and habitat information about each species. Comments, references, detailed data, and edit history are also captured to provide transparency and facilitate evaluation. Central to the SIS software is a data “calculator” that by applying the logical process described in the section above, determines the appropriate Red List categories and criteria from the species’ data entered, in particular reductions in populations, distribution, and other variables against defined parameters that influence survival. It can also calculate a range of possible categories based on uncertain or incomplete information. The user can, however, overrule any categories and criteria that have been automatically determined if so required.

IUCN standards follow a defined methodology whereby each species must be assessed within six key areas: distribution, population, habitats & ecology, use & trade, threats and conservation. The research into these six areas provides the data for determining the criteria to which one of the nine categories may be assigned:

#### Distribution and location

There are two measures of the spatial spread relevant to the assessments together with an indicator of event occurrences (location) that could result in extinction:

a) Extent of Occurrence (EOO) is a minimum convex polygon drawn around the species’ range that may include unsuitable habitat such as land, as well as deep water across which larvae may disperse but unsuited for settlement, and

b) Area of Occupancy (AOO) which is the sum of the areas of habitat in which the species is known to occur. For shallow water species, including the majority of haliotids, the AOO may be calculated from the length of coastline in which the species occurs extended by the width of habitat along its bathymetric range, or, for these mostly linear habitats, standard 4 km^2^ (2 km by 2 km) cells as suggested in IUCN standards [55].

Location. Species that occur within a restricted EOO or AOO are at greater risk from catastrophic events. The ‘location’ count indicates the number of areas in which a single catastrophic event, for example a MHW or oil spill from adjacent refinery, could affect all individuals of the taxon present and cumulatively drive a species into extinction. Distribution and location are both key factors in determining extinction risk to species of restricted range.

#### Population

Measurements of population size may indicate temporal variations in abundance and the probability of species decline leading to extinction. For cryptic species and those in inaccessible habitats such as deep water, this may be difficult to determine. For the majority of abalone species, predominantly those of no commercial interest, the literature is silent on population sizes, however, for species that are commercially fished, data are frequently available from the regulating authorities for establishing baseline abundance / density or to determine a total allowable catch (TAC) for stock management. These normally include separate quotas for recreational and/or Indigenous fishers. These source data, including variations in tonnage landed and, more rarely, catch per unit effort (CPUE) can act as proxies for determining the rate of change in a population [56]. One caveat: with highly aggregated species such as abalone, CPUE may be hyperstable and a poor predictor of declining trends up until the population crashes [57, 58]. Fishery data however, by their nature, are unable to accurately quantify illegal, unreported and unregulated (IUU) fishing that continues to blight many abalone fisheries worldwide [54, 59].

#### Habitats and ecology

Abalone larvae require crustose coralline algae (CCA) on which to settle and for early development, before migrating onto algal beds to sustain them in their adult lives [48]. Natural and manmade threats including seaweed harvesting, pollution, MHWs, and herbivorous intruders, can modify the natural ecology and result in semi-sessile taxa such as abalones being expurgated. Sea urchin barren grounds for example, render areas that once supported abundant abalone populations devoid of algae and no longer able to sustain them resulting in a diminishing AOO [21].

#### Use and trade

Abalones have been fished by Indigenous peoples for millennia and hold special religious and cultural significance. Many local coastal communities have long traditions of subsistence fishing, a touchstone for community and family social events [60, 61]. Abalones are a valuable commodity, sought after for both their meat and shells and much prized as a delicacy. High prices ensure there is a ready market for both legally and illegally fished animals, with poaching to some degree a recurrent issue within almost all abalone fished areas [54]. Although most fisheries are governed by regulation, enforcement is often patchy and it is common for quotas to be exceeded or for closed fishery areas to be ignored. In addition to licensed commercial fisheries, active recreational fisheries will often be separately regulated with bag limits, numbers held and restrictions on resale.

#### Threats

As may be seen in the regional assessments below, abalones are faced with a multiplicity of threats, exacerbated by the biological requirement for individual animals to be in close proximity for successful reproduction [45]. General threats include poaching and overfishing, habitat loss, MHWs, diseases including viruses, competition from sea urchins including kelp forest transitions to sea urchin dominated systems, pollution and predation. Withering Syndrome, a fatal bacterial disease first observed in 1985 in black abalone (*H*. *cracherodii*) of California [62], has since spread to other abalone species initially within the same locality but subsequently through trade to other regions of the world, infecting both wild and cultured animals with mortality reported of up to 99% [26]. For the disease to be expressed, an infected abalone must be exposed to warm waters such as during an El Niño event to cause mortality [62, 63]. Future threats also include ocean acidification and general warming of the oceans from the combustion of fossil fuels. As shallow water species, warming seas can also have a direct impact on the primary food resource, algae.

#### Conservation

There are many instances of conservation for species that have suffered from the effects of overfishing and the decline in abundance. To address these declines, restoration programmes have been established, including MPAs, translocation of adults and captive breeding programmes. With the abalone aquaculture industry established in many countries, partnerships between aquaculture and fisheries in the form of restoration mariculture may help bolster wild stocks and could be a critical feature for future conservation efforts [64, 65].

### Synthesis and pre-publication checks

Following our research and assessment, the results were reviewed by an international group of species and subject matter experts acting in a peer-review capacity, with each having a specialised knowledge of those species they assessed. Owing to Covid-19 travel restrictions, the reviews were primarily conducted by email and video conferencing in place of the customary IUCN workshop scenario. The commercially fished species were reviewed by specialists both from within academia and also from government fishery authorities who undertake abalone stock assessments, establish catch quotas and liaise with anti-poaching enforcement. This peer-review process confirmed or modified findings of the original assessors and allowed inclusion of supplementary field-based knowledge from the participating experts. All reports were checked for consistency by the Mollusc Specialist Group of the IUCN Species Survival Commission before final approval and submission for publication through the IUCN Red List Unit. The assessments were published by IUCN in two tranches in the years 2021 and 2022.

## Results

### Overview

Abalones can generally be classified into two groups–those that are (or have been) fished commercially and those that are generally not exploited owing to their small size. Although lacking data, some of the small non-commercial species may also be taken incidentally as food, but this would be through gleaning by local subsistence fishers and likely to have only a marginal effect on populations, unlike organised fisheries including recreational abalone fishing. Furthermore, while poaching has had a catastrophic impact on some commercial fisheries it is likely negligible on unexploited species.

**Fig 1:**
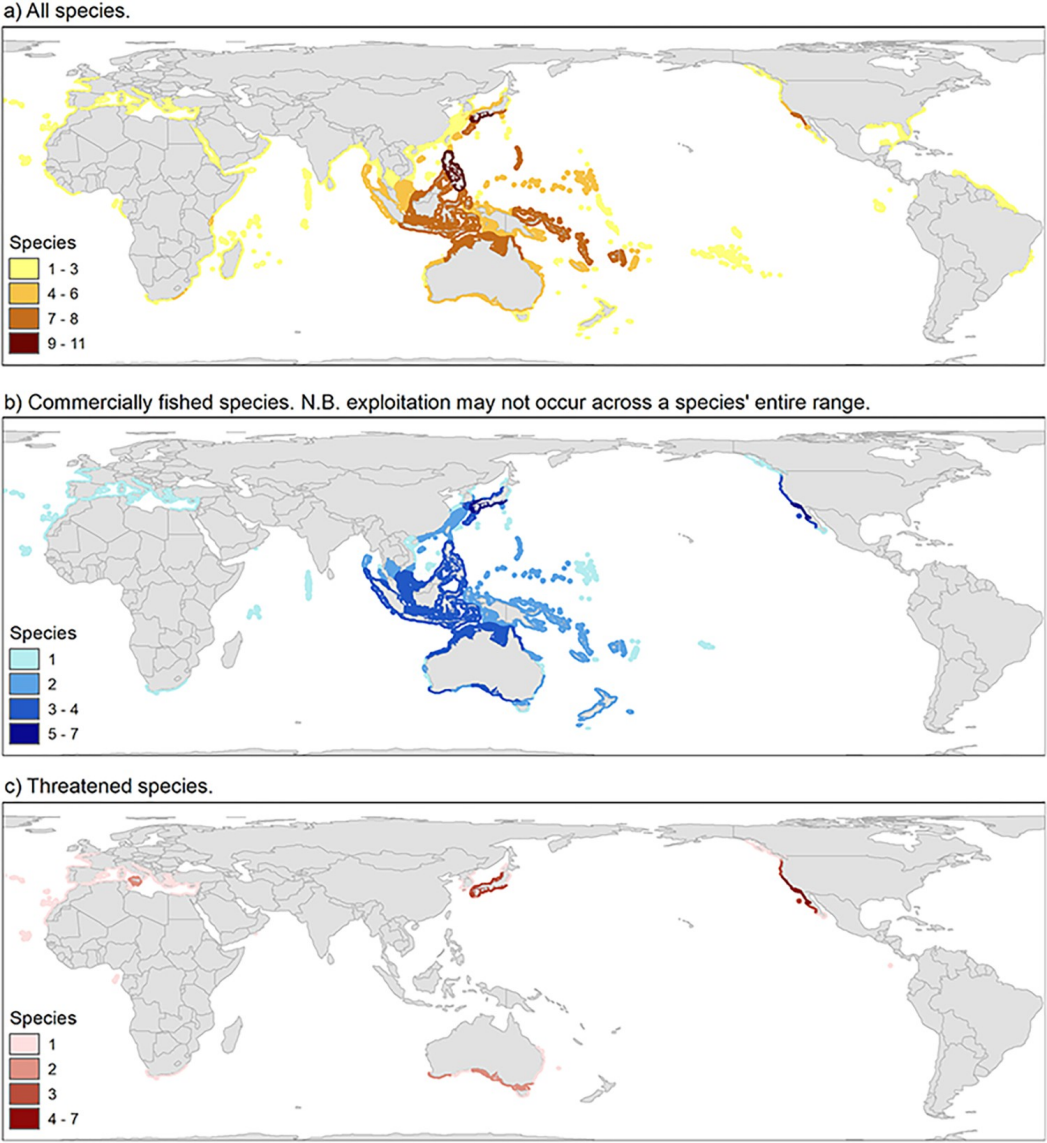
Global distribution of abalone spp. (a) all species, (b) species commercially fished, and (c) species threatened with extinction—Critically Endangered (CR), Endangered (EN) or Vulnerable (VU). Basemap (i.e. country layer only) sourced from Natural Earth. Free vector and raster map data @ naturalearthdata.com.

A digital distribution map accompanies each IUCN Red List species assessment. Maps were generated in ArcGIS (Environmental Systems Research Institute) based on occurrence records primarily following Geiger & Owen (2012) and reviewed by species experts [34]. To improve visualization at a global scale, maps were standardized using a base map for nearshore coastal species that extends to either the 200 m bathyline or 100 km from shore. For species of restricted range, the area within which the species occurs and whether it is contracting or becoming fragmented can be an important indicator in assessing extinction risk. From these species maps and for this summary, global maps of overall species richness, commercially fished species, and threatened species were generated in ArcGIS 10.8. For each of the three analyses, distribution maps were overlaid to determine the number of species occurring in each 100 km^2^ grid cell. Fig 1 shows the global distribution of a) all species (n = 54), b) commercially fished species (n = 21) (note, some species may not be fished across their entire distribution, especially where local regulations may include a size limit, or gear restrictions e.g., no SCUBA), and c) threatened species only (n = 20).

**Fig 2:**
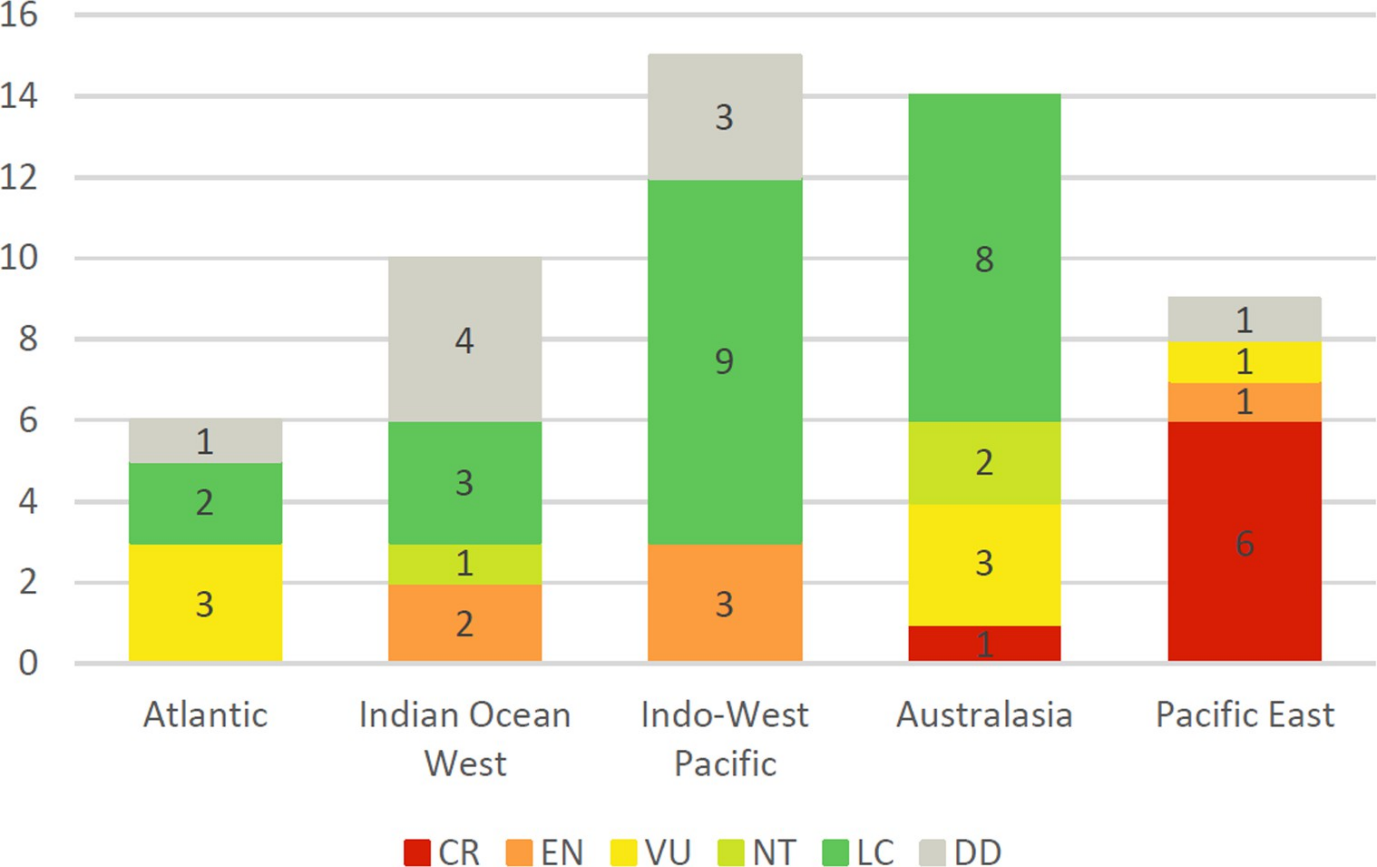
Numbers of abalone species by category code analysed by oceanic region.

Of the 54 species of abalone assessed globally, 20 (37.04%) are threatened with extinction, i.e., categorised as Critically Endangered (CR), Endangered (EN) or Vulnerable (VU), with a further three species categorised Near Threatened (NT). Fig 2 shows the number of species of each category by oceanic region. For further details refer to Tables 3 to 7 attached to regional analyses.

**Table 3:** Atlantic abalone.

Species name	Size	C	Distribution	Cat
	**mm**		**Atlantic**	
*H*. *geigeri*	41		São Tomé & Príncipe in the Gulf of Guinea	VU
*H*. *marmorata*	76		Senegal, Guinea, Cote d’Ivôire, Gabon	LC
*H*. *mykonosensis*	58		North-central Mediterranean, from Corsica to the Aegean coast of Turkey	LC
*H*. *pourtalesii*	31		Carolinas, Florida, NE/NW Gulf of Mexico, Cuba, Colombia, Suriname, C. Brazil	DD
*H*. *stomatiaeformis*	45		Sicily, Malta and Lampedusa	VU
*H*. *tuberculata*	135	C	English Channel Is. S. to Northwest Africa (Mauritania) and the Mediterranean	VU

‘C’ denotes commercially fished.

**Table 4:** Abalone species of Western Indian Ocean.

Species	Size	C	Distribution	Cat
	**mm**		**Western Indian Ocean**	
*H*. *alfredensis*	79		Pondoland (Mbotyi) in NE Transkei to Port Alfred on Eastern Cape, South Africa	DD
*H*. *arabiensis*	40		N Salalah, Oman to N. Fujairah, UAE	NT
*H*. *mariae*	144	C	S Oman (Dhofar) between Mirbat and Hassik	EN
*H*. *midae*	234	C	N. Port St Johns on Eastern Cape to St. Helena Bay, Western Cape, South Africa	EN
*H*. *parva*	59		Coffee Bay on the Eastern Cape of South Africa to False Bay, Cape Town	DD
*H*. *queketti*	52		NE Somalia to Transkei, South Africa, also Mozambique—other reports questionable	DD
*H*. *rugosa*	63		Yemen and Red Sea to Park Rynie, South Africa, also Mascarenes and Madagascar	LC
*H*. *spadicea*	105		Northern KwaZulu-Natal to Partridge Point, Cape Peninsula, Western Cape, S. Africa	LC
*H*. *squamosa*	103		Madagascar: Lavanono to Fort Dauphin (Taolagnaro)	DD
*H*. *unilateralis*	40		N Red Sea and Oman to Eastern Cape of S. Africa and east to Mascarene Islands	LC

‘C’ denotes commercially fished.

**Table 5:** Abalone species of Indo-West-Pacific.

Species	Size	C	Distribution	Cat
	**mm**		**Indo-West-Pacific**	
*H*. *asinina*	120	C	Andamans, Japan, Malaysia, Indonesia, S. China, Viet Nam, Philippines, Australia, Fiji	LC
*H*. *clathrata*	46		E. Africa, entire Indo-West-Pacific incl. Japan, Australia, and E. to Tonga.	LC
*H*. *discus*	232	C	Japan and Korea	EN
*H*. *dissona*	36		Australia (Queensland), Fiji, Guam, New Caledonia, N. Mariana Is, Tonga	LC
*H*. *diversicolor*	109	C	Southern Japan, China, and across much of Southeast Asia to northern Australia	DD
*H*. *fatui*	75		Indonesia (Papua), N. Mariana Is, Philippines, Solomon Is, Tonga	DD
*H*. *gigantea*	233	C	Japan and Korea	EN
*H*. *glabra*	68		Japan, Malaysia, Indonesia, Philippines, N. Australia	LC
*H*. *jacnensis*	30		Japan, Philippines, Indonesia, New Caledonia, Micronesia, Tonga, Am. Samoa, Nieu	LC
*H*. *madaka*	245	C	Japan and Korea	EN
*H*. *ovina*	92	C	Maldives to Society Is.; SW Japan to Queensland and Western Australia	LC
*H*. *papulata*	27		Sri Lanka, Philippines, Thailand, Papua New Guinea, N. Australia	LC
*H*. *planata*	50		Sri Lanka to Fiji and from Japan to N. of W. Australia	LC
*H*. *pulcherrima*	45		French Polynesia incl. Tuamotus, Society Is, Gambier Is, and Marquesas; Pitcairn Is	DD
*H*. *varia*	86		Sri Lanka to Tonga and from Japan to Australia	LC

‘C’ denotes commercially fished.

**Table 6:** Abalone species of Australasia.

Species	Size	C	Distribution	Cat
	**mm**		**Australasia**	
*H*. *brazieri*	50		Mooloolaba, S. Queensland to Jervis Bay, NSW, poss. to Wilson’s Promontory, Vic.	NT
*H*. *coccoradiata*	80		S. of Brisbane, S. Queensland to Cape Conran, Victoria, poss. to SE of Melbourne	LC
*H*. *cyclobates*	97		Esperance, WA to Wilson’s Promontory, Victoria	LC
*H*. *elegans*	120		Esperance, north to the S. passage of Shark Bay, WA	LC
*H*. *laevigata*	230	C	Geographe Bay S. of Perth, WA to Victoria/NSW border; N. coast of Tasmania	VU
*H*. *melculus*	56		Keppel Island, Queensland to the border with NSW	VU
*H*. *roei*	139	C	Port Fairy, Victoria to Shark Bay, WA	NT
*H*. *rubiginosa*	56		Lord Howe Island, NSW	CR
*H*. *rubra*	248	C	NSW, Victoria, Tasmania, South Australia and SW Western Australia	VU
*H*. *scalaris*	125		Kalbarri, WA to Victoria-NSW border and Tasmania	LC
*H*. *semiplicata*	75		Esperance north to Geraldton, Western Australia	LC
*H*. *australis*	110	C	New Zealand incl. Campbell Is, Stewart Is, Auckland Is and the Chatham Islands	LC
*H*. *iris*	198	C	North and South Is, Stewart Is, Chatham Is, The Snares Is and Three Kings Is.	LC
*H*. *virginea*	75		All of New Zealand and outlying islands	LC

‘C’ denotes commercially fished.

**Table 7:** Abalone species of Eastern Pacific.

Species	Size	C	Distribution	Cat
	**mm**		**Eastern Pacific**	
*H*. *corrugata*	245	C	Pt Conception, California to Baja California Sur, Mexico incl Guadalupe Is.	CR
*H*. *cracherodii*	216	C	Point Arena, California to Bahia Tortugas, Baja California Sur and Guadalupe Is	CR
*H*. *fulgens*	255	C	Point Conception, California to central Baja California Sur and Guadalupe Is.	CR
*H*. *kamtschatkana*	187	C	Sitka, Alaska south to central Baja California	EN
*H*. *rufescens*	313	C	Central Oregon to central Baja California, Mexico	CR
*H*. *sorenseni*	226	C	Point Conception, California to Central Baja California, Mexico and Guadalupe	CR
*H*. *walallensis*	179	C	Oregon to central Baja California, Mexico and San Benito Is.	CR
*H*. *dalli*	47		Galapagos Is., Gorgona Is. (Colombia), Cocos Island (Costa Rica)	DD
*H*. *drogini*	65		Cocos Island, Costa Rica	VU

‘C’ denotes commercially fished.

**Fig 3:**
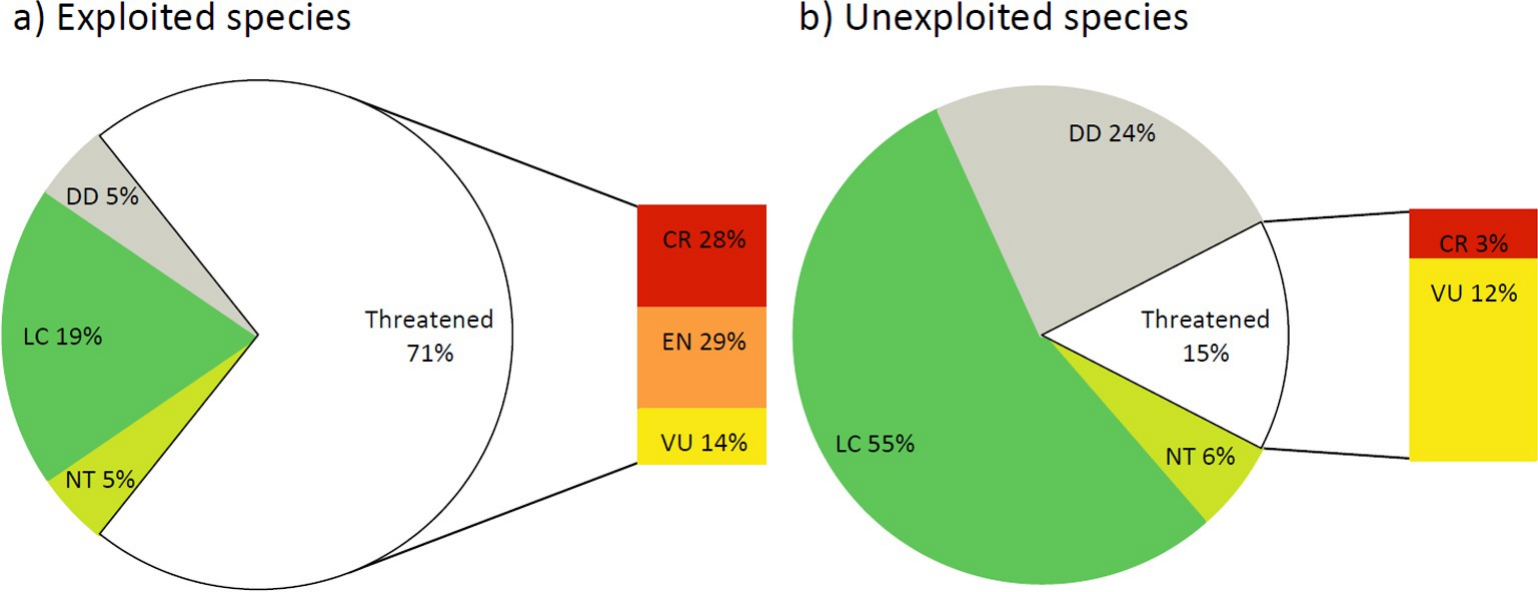
Abalone species assessed globally analysed by category. CR Critically Endangered; EN Endangered; VU Vulnerable, NT Near Threatened, LC Least Concern, DD Data Deficient, and showing the impact of commercial exploitation.

Twenty-one of all abalone species (38.89%) are (or have been) commercially fished and/or are recognised targets of recreational fishers, of which 15 (71.43%) are categorised as threatened with one further species categorised as Near Threatened (NT). Of the 33 unexploited species, only five (15.15%) are categorised as threatened with a further two as NT. Fig 3 graphically illustrates that of those species with a sufficiency of data, i.e. not categorised Data Deficient, commercially exploited abalones are over four times more likely to face extinction as unexploited species.

### Analysis of conservation status by region

In examining the abalone species by region, we divided the genus into five geographical sectors (total number of species shown in parentheses): Atlantic (6), Western Indian Ocean (10), Indo-West-Pacific (15), Australasia (14) and Eastern Pacific (9). Some wide-ranging species may spill into an adjoining sector. Threatened species occur in every region (Fig 2) but the most species listed as Critically Endangered are in the Eastern Pacific along the western coast of the United States, Canada and Mexico. The analysis described below provides a summary of the principal issues in determining their category. Comprehensive referenced data on distribution, populations and specific threats confronting each species, together with any conservation strategies that may be planned or implemented, can be found on the published IUCN Red List (iucnredlist.org) species records.

#### Atlantic

This sector includes both the Western Atlantic and the Eastern Atlantic from the English Channel south to Gabon in Central Africa, including the Mediterranean. There are six species (Table 3) of which just one, *H*. *pourtalesii* is found in the Western Atlantic.

**Fig 4:**
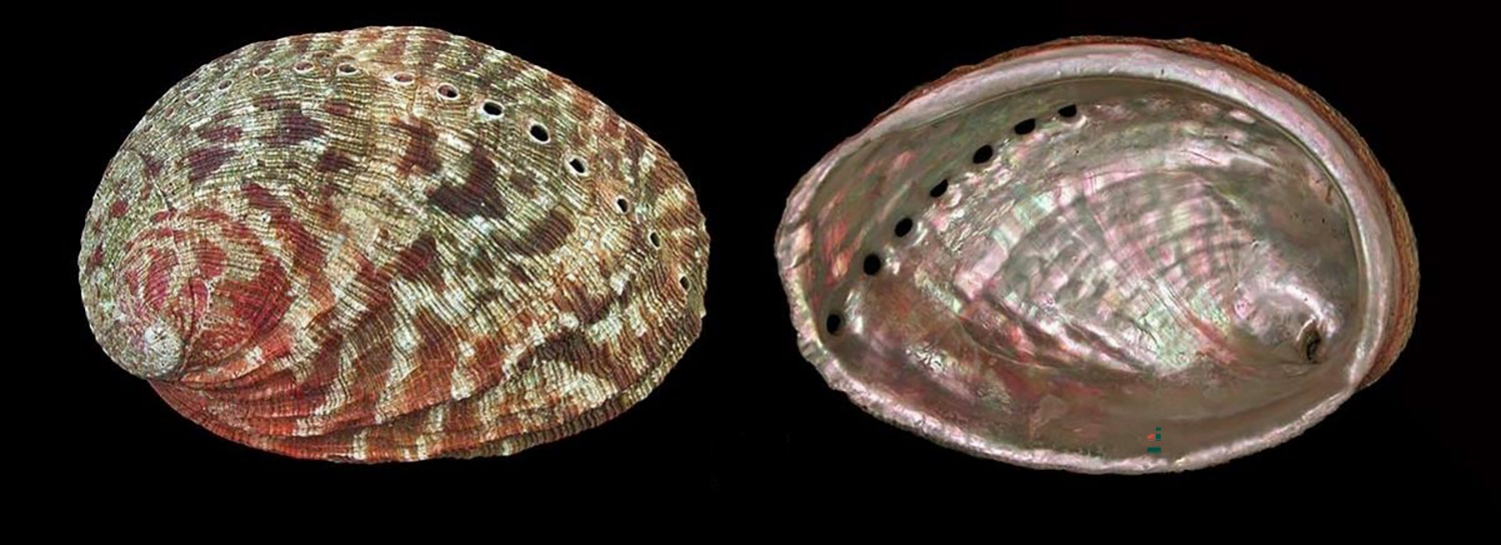
H. tuberculata (VU). Occurs in Europe & North Africa. Failure of fisheries enforcement. Reprinted under a CC BY license with permission from Buzz Owen, original copyright.

*H*. *pourtalesii* (DD) occurs south from the Carolinas to Central Brazil (including the Caribbean/Gulf of Mexico), although as it occurs in deeper waters than most abalones, its distribution is uncertain. Of the five species in the Eastern Atlantic, *H*. *tuberculata* (VU) (Fig 4), known in the UK as green ormer, is the only commercially fished species with its subspecies *H*. *tuberculata tuberculata* form *lamellosa* found throughout the Mediterranean. Although in many parts of its range this species’ fishery is controlled, failure of enforcement and poaching across large areas of habitat have considerably reduced the overall population with few signs of recovery [66]. *H*. *stomatiaeformis* (VU) only occurs in Sicily, Malta and Lampedusa where its restricted range and decline in habitat quality and the marine ecology of the region places it at risk [67]. *H*. *geigeri* (VU) occupies a highly restricted range off the islands of Sáo Tomé and Príncipe in the Gulf of Guinea, and although there are limited data on its status, it is vulnerable to ecological change, MHWs and other environmental threats [68]. The remaining two species in this sector are LC.

#### Western Indian Ocean

This sector includes the entire coastline from the Persian Gulf and Red Sea, south through East and Southern Africa including the Mascarene Islands and the whole of South Africa to St Helena Bay on the Western Cape. There are ten species (Table 4).

**Fig 5:**
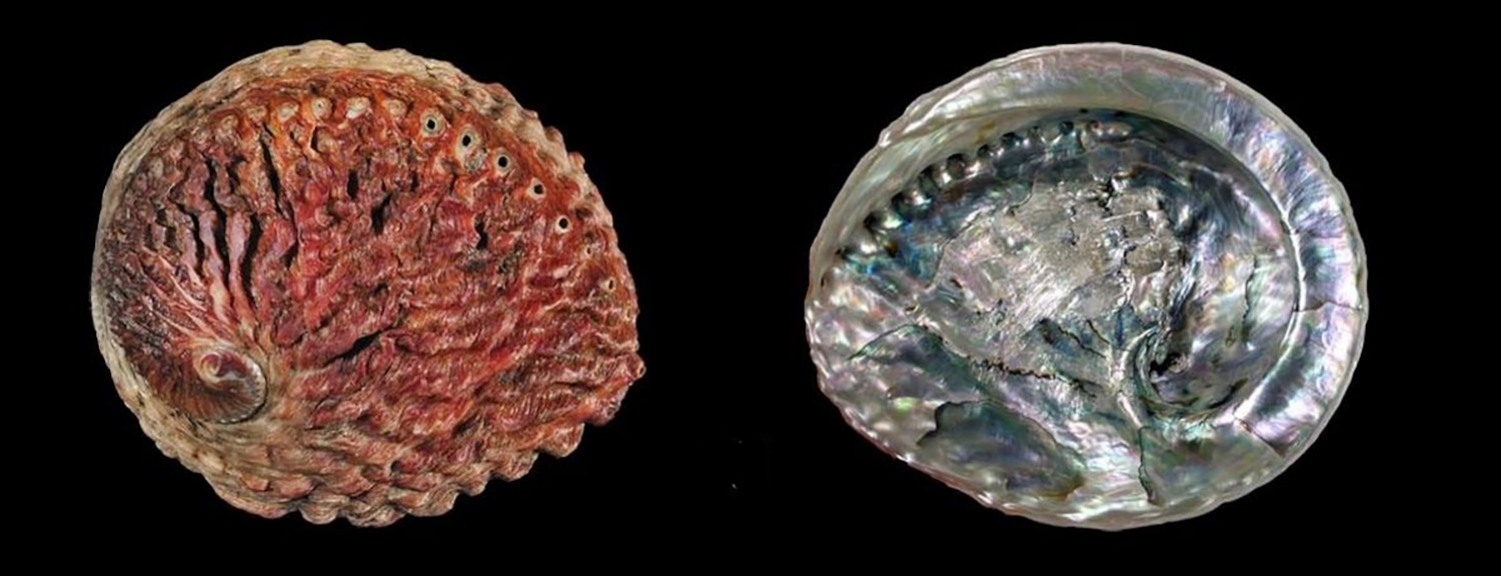
*H*. *midae* (EN). Occurs in South Africa. Victim of uncontrolled poaching. Reprinted under a CC BY license with permission from Buzz Owen, original copyright.

The recently described *H*. *arabiensis* Owen, Regter & Van Laethem, 2016 (NT) has a highly restricted range in southern Oman and is considered very rare. *H*. *mariae* (EN), known as the Omani abalone, is the only commercially fished species within this group occurring outside of South Africa. Continuing over-exploitation coupled with pollution, weather and harmful algal blooms have extirpated populations across a third of its distribution [69, 70]. *H*. *queketti*, *H*. *squamosa*, *H*. *alfredensis* and *H*. *parva* (all DD) suffer a paucity of data on populations with some distributions unresolved. *H*. *midae* (EN) (Fig 5), the most important commercially fished abalone in Africa and commonly known as ‘perlemoen’, has for many years been subject of a commercial fishery, recreational fishing and uncontrolled poaching by organised criminal gangs, often linked to the narcotics trade, driven by high consumer demand [71, 72]. The managed fishery has been almost reduced to a footnote leading to widespread species recruitment failure. Although in 2007 this species was listed by CITES, it was withdrawn three years later owing to implementation challenges [72]. More recently there have been some successful attempts at stock enhancement from hatchery-produced seed introduced into kelp beds outside their natural distribution (abalone ranching) [73]. All other species in this sector are of LC.

#### Indo-West-Pacific

This sector includes all of the Indian Ocean except its western rim, together with Southeast Asia, East Asia and the islands of the Western Pacific. It also includes species that are non-endemic to Australia that occur there. There are 15 species (Table 5).

**Fig 6:**
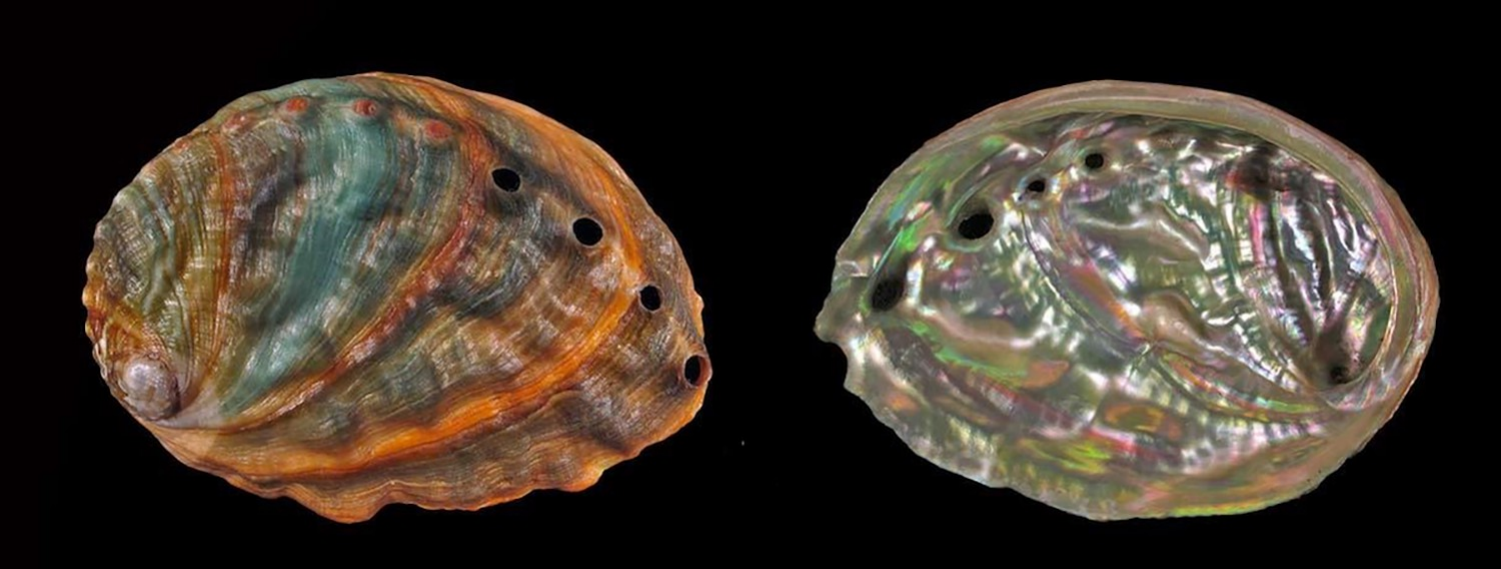
*H*. *discus hannai* (EN). Occurs in Japan & Korea. The world’s most consumed abalone. Reprinted under a CC BY license with permission from Buzz Owen, original copyright.

Nine species are small to medium sized and widely distributed in tropical waters where they are not subject to mass exploitation and are classified as LC. However, two of these LC species, *H*. *ovina*, commonly called the sheep’s ear abalone and *H*. *asinina*, the donkey’s ear abalone, are commercially fished with some consideration given towards aquaculture, although their small size is generally not as attractive to the important East Asian market. *H*. *fatui* Geiger, 1999 (DD) is a recently described species for which there is insufficient data on its true distribution or on population size on which an assessment may be made. *H*. *pulcherrima* (DD) is only known from empty shells despite its wide distribution across French Polynesia and the Pitcairn group. The remaining five species in this group are all fished commercially. *H*. *discus* (EN) with its subspecies, *H*. *d*. *hannai* (Fig 6) is the most extensively cultured species of abalone in the world, with major facilities in Japan, South Korea and Northern China as well as outside its natural distribution in places such as Chile, although here largely superseded by red abalone (*H*. *rufescens*) [74]. Despite the success of captive breeding for wild stock replacement, populations are in steep decline as a result of overfishing and the loss of habitat, in particular, its preferred species of brown macroalgae [75]. Its high retail value makes this species a target for poaching; although enforcement has been strengthened in Japan with severe penalties, it remains a serious issue [76]. *H*. *gigantea* (EN) has historically been consolidated with *H*. *madaka* (since accepted as a separate species), and there are no long-term unambiguous data on *H*. *gigantea*. Research into populations at its southern extent indicates extirpation caused by local loss of algae [77]. Although *H*. *gigantea* occurs sympatrically with *H*. *d*. *discus* and *H*. *madaka*, the total catch of *H*. *gigantea* alone is not distinguishable. It has been calculated that the total annual catch of all three species combined has declined dramatically [78]. *H*. *madaka* (EN) was until recently considered a synonym of *H*. *gigantea* (see above), and data of both species are therefore confused. Because *H*. *madaka* has been severely overfished for many years, it is now extremely scarce in the overall abalone catch in Japan, with research determining that in one area, landed values decreased year on year, with no specimens landed after 2009 [78]. It has also suffered the consequence of anti-fouling chemicals used on vessel hulls [79]. The collapse in combined stocks of *H*. *discus*, *H*. *madaka* and *H*. *gigantea*, although inseparable by species, would indicate that one or more may have already passed the threshold for Critically Endangered, and all would benefit from comprehensive empirical evidence. Although *H*. *supertexta* is considered by WoRMS and Geiger & Owen (2012) to be a separate species [34], Hsu & Gwo (2017) using molecular markers determined it to be the subspecies, *H*. *diversicolor supertexta* [80]. Owing to this long-standing taxonomic confusion, *H*. *diversicolor* has been categorised as DD. All other species in this sector are of LC.

#### Australasia

This sector includes endemic species only occurring in Australia and New Zealand together with their islands. There are 14 species (Table 6).

**Fig 7:**
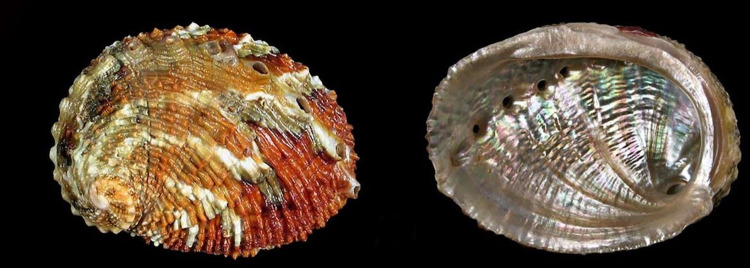
*H rubiginosa* (CR). Occurs in Lord Howe Island, NSW. The only critically endangered unexploited species. Reprinted under a CC BY license with permission from Buzz Owen, original copyright.

To fish for abalones in Australian waters, the animal must be of a minimum size, and although some specimens of *H*. *elegans* (LC) and *H*. *scalaris* (LC) are large enough for human consumption, they fail to conform to these regulations and cannot be legally fished. Many species suffer from increasingly frequent MHWs, including *H*. *brazieri* (NT), a small, scarce species with a fragmented population [81], and *H*. *melculus* (VU), an extremely rare species that occurs along the Sunshine Coast of southern Queensland [82], an area zoned for shoreline development, including artificial reef construction. The resultant sediment transport is an ongoing threat to this species’ shallow water habitats throughout its range [83]. *H*. *laevigata* (VU), known as greenlip abalone, although subject of a well-managed commercial fishery, has seen a decline in biomass as a result historic fishing pressure, MHWs in the west of its range, together with poaching [84]. Of eight fishery zones across four jurisdictions, none is currently classified as sustainable [85]. *H*. *roei* (NT), is the smallest of the three commercially fished Australian abalone and is just 2% of the total take. The majority occurs in Western Australia where a MHW in 2011 decimated stocks inflicting 99% mortality in its most northerly, i.e., warmer reaches, the effects of which are still reflected in its overall abundance [86]. There is a robust management plan for its recovery but the increasing incidence of MHWs point to a problematic future. *H*. *rubra* (VU) has two subspecies: *H*. *rubra rubra*, known as blacklip abalone and *H*. *rubra conicopora* known as brownlip abalone with the latter restricted to the southwest of Western Australia. Blacklip abalone comprises 80% of the total Australian abalone catch. Of 14 fishery zones across four states, eight are classified as sustainable, two are depleting, two are depleted and two are undefined through lack of data [87]. Although this is now a well-regulated and managed fishery, this species is also exposed to the effect of MHWs and has also been subject to viral infection. Abalone viral ganglioneuritis (AVG), introduced into the marine environment from an aquaculture facility located in Western Victoria, resulted in up to 90% mortality in down-current waters, and although the threat is now considered low, it is an ongoing concern [88]. *H*. *rubiginosa* (CR) (Fig 7) is endemic to Lord Howe Island in the Tasman Sea east of Port Macquarie, NSW. The island is small, just 10 km in length. Because of this species’ rarity, highly restricted range and exposure to MHWs, it has been assessed as CR. In New Zealand, there are three abalone species, known as pãua, two of which are exploited, and although the smaller *H*. *australis* (LC) was once fished on a commercial basis, the fishery is now closed and it is only taken by recreational fishers for which there are enforced daily bag limits and size restrictions. *H*. *iris* (LC), known as blackfoot pãua, is the only active commercial abalone fishery in New Zealand in which it is fished for food, used in jewellery and raised in hatcheries for pearls. It is also subject to strict fishery controls, where the total allowable commercial catch (TACC) is based on population trend models largely influenced by catch per unit effort (CPUE), with all recent data suggesting that the stock has been improving since the mid-2000s [89]. Since 2010, the use of dive loggers has been steadily increasing, enabling fine-scale monitoring which should allow complex metrics such as spatial CPUE. All other species in this sector are LC.

#### Eastern Pacific

This sector includes all abalone species occurring along the western seaboard of the USA, Canada and Mexico together with the only two species found at islands off Central and South America. There are nine species (Table 7).

**Fig 8:**
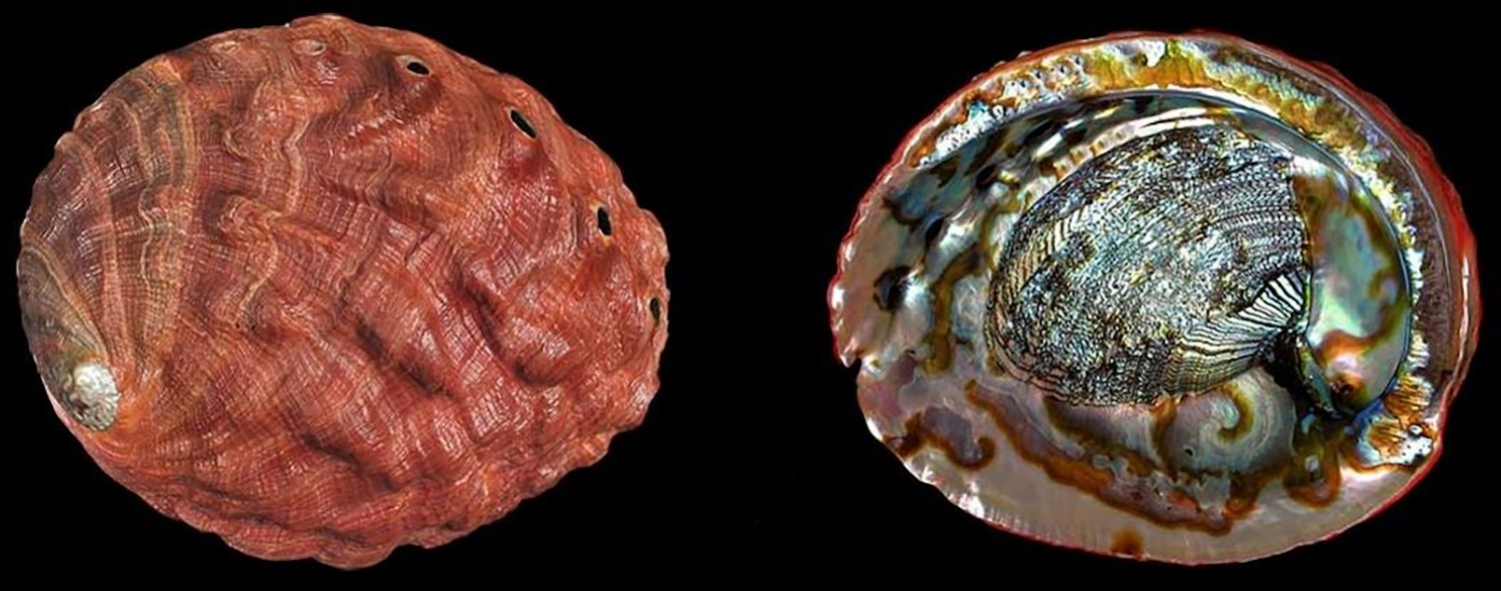
*H*. *rufescens* (CR). At 313 mm, the world’s largest abalone Reprinted under a CC BY license with permission from Buzz Owen, original copyright.

Of the two Eastern Pacific species that occur outside North America, *H*. *dalli* (DD) is a small, rare, deep-water species from the Galápagos Islands and also Gorgona Island off the Pacific coast of Colombia, with a separate subspecies occurring off Cocos Island, Costa Rica. There are few data on its ecology. *H*. *drogini* (VU), endemic to Cocos Island, although also suffering a paucity of data owing to its highly restricted range, is vulnerable to catastrophic events such as MHWs. Of the remaining Eastern Pacific abalone, all but one are listed as CR, including the white abalone (*H*. *sorenseni*) (CR) which is on the U.S. Endangered Species List and on the verge of extinction in the wild [5]. This species now has a well-established recovery programme including a captive breeding component which has been in the making for nearly 20 years when the first wild white abalone were captured for conservation aquaculture. Today, thousands of juveniles have been produced and a robust partnership has been developed with federal, state, universities and private partners [65], leading to an annual restocking programme begun in 2019, but with abundant predators in the region and a loss of kelp through MHWs, reseeding has been challenging and remnant populations continue to decline. Black abalone (*H*. *cracherodii*) (CR) was also overfished and then suffered mass mortalities owing to Withering Syndrome, a disease triggered when abalone testing positive to the causative bacteria are exposed to warm water events. Today, there are some hopeful signs of recruitment in some areas being reported, but other areas, including Mud Creek in central California, have been hit by landslides occurring when heavy winter rains fall in fire-scarred areas of the central California coastline. Pink abalone (*H*. *corrugata*) (CR) continues to be at low densities in California [90] compared to the 1970s when it was a dominant species in the fisheries. Subsequent to the southern California fisheries closure in 1997 there has been persistent recruitment failure resulting from the Allee Effect. Green abalone (*H*. *fulgens*) (CR) is still uncommon in most locations after more than 24 years of fishery closure, but is making a comeback in very few locations in southern California such as Santa Catalina Island [91] although their spatially restricted rebound is a concern for their vulnerability. Flat abalone (*H*. *walallensis*) (CR) is also close to extinction in the wild. In the past, this species was always low in abundance with a very narrow distribution [10]. Today it has disappeared from the Monterey area and recent surveys (2022) in northern California, a former stronghold of the species, resulted in no flat abalone sightings (Rogers-Bennett pers. observ.). Northern or pinto abalone (*H*. *kamtschatkana*) (EN) in contrast has a wide geographical distribution and, in some regions, such as British Columbia, are numerous with signs of recruitment with juveniles greater than 20 mm [92]. This species has two morphologies with *H*. *kamtschatkana kamtschatkana* or pinto abalone (aka northern abalone) in the north and *H*. *kamtschatkana assimilis* or threaded abalone in the south of its range. In southern regions, it is less abundant, and populations continue to decline. In Washington State, there is an active restoration programme and juveniles produced in hatcheries are stocked in the wild, where they are showing good signs of growth and survival, particularly at some restoration sites [93]. Red abalone (*H*. *rufescens*) (CR) (Fig 8), once the foundation of the commercial abalone fishery, was extirpated in central California owing to a combination of fishing and sea otter predation but remained for a time abundant in southern California until their decline due to overfishing, forcing the closure of all abalone fisheries in the south in 1997 [13]. There had been a robust free diving (only) recreational fishery in northern California worth an estimated $44 M with more than 31 000 participants per year [94]. The fishery had many sustainability management measures in place including size limits, seasonal closures, daily and annual bag limits as well as gear restrictions including no SCUBA. From 2014–2016 a massive MHW decimated the bull kelp (*Nereocystis luetkeana*) forests and sea urchin populations increased, dominating the system leading to mass abalone mortalities. Rapid declines by more than 80% of the red abalone [21, 52] led to the closure of this, the last open abalone fishery in the United States in 2018 (with the exception of small-scale subsistence fisheries for pinto abalone in SE Alaska).

## Discussion

Our analysis clearly shows that abalone species exposed to fishing have a reduced resilience and a significantly greater chance of extinction compared to those that are unexploited. The few unexploited species assessed as threatened are generally characterised by occurring within a highly restricted range where MHWs and loss of habitat could drive an extinction event. Although overfishing for abalones can be observed through an historical lens, the impact is felt to this day in fisheries around the world where widespread stock declines have left remaining populations more vulnerable to a suite of stressors [53]. Most fisheries nowadays are subject to management controls ranging from total closure to enforcement of quotas, mandatory data loggers and the banning of SCUBA. Management measures that restrict catch provide greater protection of minimum abalone densities necessary for successful fertilization. These include bag and size limits, permanent MPAs to protect identifiable populations, and prohibition of SCUBA for deep stock conservation, rather than seasonal closures which simply shift catch effort to later seasons. Monitoring stocks using fishery independent surveys provides baselines estimates for comparisons following major environmental events [95]. Ideally, this should include kelp and algal cover, as well as competitors such as sea urchins, and also predators that can, when out of balance, severely impact abalones. Pre-impact data can also inform managers of the causes of any subsequent population declines, including identification of harmful algal blooms [96] that also devastate farmed abalones [97].

Although wild abalone fisheries have declined in most regions globally, with estimated 1970s landings of 20 000 tonnes reduced to 4500 tonnes by 2020/21 [54], the commercial loss has been more than replaced by an upscaling in aquaculture, where total global production in 2010 of 65 344 tonnes had, by 2020/21, reached 243 506 tonnes. Aquaculture production is dominated by China with 89% of the total, followed by Korea with 8%, South Africa at 1% and ten other countries each below one percent [54]. Regrettably, this explosive development has not seemingly reduced the pressure on wild populations which continue to be fished, often to unsustainable levels. Nevertheless, aquaculture offers a bonus in sourcing seed for augmenting depleted wild fisheries, enabling ocean ranching and encouraging population recovery [98], although reintroduction programmes demand complex and costly stock management and record-keeping to safeguard the genetic diversity of wild populations. Heasman et al. (2007) suggest a minimum number of parents at 30 to 40 of each sex, with a large number of mating pairs over each successive generation to avoid inbreeding problems [98].

While this partnership between aquaculture and wild fisheries can appear promising, especially in the face of climate driven stressors, greater support and funding by foundations and governments is still needed. Despite this exponential increase in abalone aquaculture, the decline in wild populations persists while success to reverse it can only be described as modest with current levels of support. Even with sustainable fisheries, failures in policing and enforcement can rapidly reverse gains where there is aggressive and widespread poaching, especially from organised criminal gangs. It may be the case that effective visible protection of large wild abalone assemblages will be more successful and less costly than relying on restocking with aquaculture produced abalones [99]. Recent work has demonstrated the importance of sustainable fishing, conservative fishing quotas and periodic fishery closures. While protecting larger females within MPAs that can serve as climate refugia, bolstering resilience to climate stressors will require close collaboration between fishers, fishery managers and scientists [100].

Elevated sea-surface temperatures are giving rise to a multiplicity of immediate threats through loss of algae and habitat, expansion of pathogens and transition of marine ecosystems. Acidification of the oceans, resulting from the current levels of absorption of CO_2_ from the atmosphere, will have a deleterious effect not only on the development of larval shells but also on the crustose coralline algae (CCA) on which larval settlement depends [14, 101, 102]. However, it should be noted that while CCA may have reduced growth and survival during acidification, it has been recorded that elevated pCO_2_ in upwelling acclimated waters maintained settlement cues for *H*. *rufescens* after prolonged acidification exposure [103]. Furthermore, low oxygen conditions in a warming ocean will also impact growth and survival of abalones [104]. Evidence shows that over future decades, abalones, in common with most, if not all calcareous marine taxa, will become increasingly vulnerable to such changes in ocean chemistry. In addition to the impact on calcification, fertilisation, embryonic larval development and settlement may all be compromised [105] allowing for less surplus productivity for fisheries.

We know the environment for abalones will in future be warmer and less oxygenated as MHWs are predicted to increase in frequency, intensity and duration [106]. Anomalous heating at the ocean surface is driven by changing climate and further influenced by El Niño-Southern Oscillation (ENSO). ENSO events result in the episodic warming and cooling of the tropical Pacific, and although this effect is centred on the eastern Pacific, its influence can be observed across the whole of the Pacific, Atlantic and Indian Oceans [107]. During the austral summer of 2010/11 an extreme MHW affecting 2000 km of Western Australia coastline resulted in 99% mortality of Roe’s abalone (*H*. *roei*) [108]. Such MHWs not only cause mortality of sedentary taxa such as abalones, but the algae on which they survive. Bull kelp (*Nereocyctis luetkeana*), the principal diet of the red abalone (*H*. *rufescens*), is usually common off the northern California coast but declined by more than 90% following the 2014–2015 MHW [109]. This decline in kelp was aggravated by an explosion in population of the purple sea urchin (*Strongylocentrotus purpuratus*) along with the extirpation from sea star wasting syndrome of the sunflower star (*Pycnopodia helianthoides*) [110], an important urchin predator in kelp forest ecosystems [21]. This resulted in 80% mortality of abalones in Northern California and the closure in 2018 of the valuable, popular and culturally important recreational red abalone fishery. Similarly, low oxygen events are impacting abalones in Baja California, Mexico [111]. Unless the combustion of fossil fuels that is driving temperature and acidification is arrested, the long-term future of abalones together with all other marine molluscs remains uncertain. We will need to use all the tools at our disposal including restoration aquaculture, reductions in fishing pressure, MPAs [112], favourable microclimates [113] and genetically more resilient families [114] to support abalones given that we know how vulnerable these molluscs are to climate stressors. They are truly oceanic “canaries in the coalmine”.
